# An Adverse Outcome Pathway for food nanomaterial-induced intestinal barrier disruption

**DOI:** 10.3389/ftox.2024.1474397

**Published:** 2024-12-24

**Authors:** Deborah Stanco, Dorelia Lipsa, Alessia Bogni, Susanne Bremer-Hoffmann, Laure-Alix Clerbaux

**Affiliations:** ^1^ European Commission, Joint Research Center (JRC), Ispra, Italy; ^2^ Independent Researcher, Ispra, Italy; ^3^ Institute of Experimental and Clinical Research, UCLouvain, Brussels, Belgium

**Keywords:** food, nanomaterial, adverse outcome pathway, endocytosis, intestinal barrier disruption

## Abstract

**Introduction:**

The ingestion of nanomaterials (NMs) may impair the intestinal barrier, but the underlying mechanisms remain evasive, and evidence has not been systematically gathered or produced. A mechanistic-based approach would be instrumental in assessing whether relevant NMs disrupt the intestinal barrier, thereby supporting the NM risk assessment in the food sector.

**Methods:**

In this study, we developed an adverse outcome pathway (AOP) based on biological plausibility and by leveraging information from an existing NM-relevant AOP that leads to hepatic outcomes. We then extracted the current evidence from the literature for a targeted selection of NMs with high relevance to the food sector, namely, ZnO, CuO, FeO, SiO_2_, and Ag NMs and nanocellulose.

**Results:**

We propose a new AOP (AOP 530) that starts with endocytic lysosomal uptake, leading to lysosomal disruption inducing mitochondrial dysfunction. Mitochondrial impairments can lead to cell injury/death and disrupt the intestinal barrier. The evidence collected supports that these food-related NMs can be taken up by intestinal cells and indicates that intestinal barrier disruption may occur due to Ag, CuO, and SiO_2_ NMs, while only few studies support this outcome for FeO and ZnO. Lysosomal disruption and mitochondrial dysfunction are rarely evaluated. For nanocellulose, none of the studies report toxicity-related events.

**Conclusion:**

The collection of existing scientific evidence supporting our AOP linking NM uptake to intestinal barrier impairments allowed us to highlight current evidence gaps and data inconsistencies. These inconsistencies could be associated with the variety of stressors, biological systems, and key event (KE)-related assays used in different studies. This underscores the need for further harmonized methodologies and the production of mechanistic evidence for the safety regulatory assessment of NMs in the food sector.

## 1 Introduction

### 1.1 Regulatory needs in the risk assessment of food nanomaterials

Nanotechnology presents many possibilities for the food industry, offering potential benefits like targeted nutrient delivery, improved food preservation, and enhanced sensory experiences. However, due to their unique properties at the nanoscale, nanomaterials (NMs) might necessitate additional testing requirements. To implement the information requirements, the European Food Safety Authority (EFSA) has published two guidance documents outlining the criteria for evaluating materials at the nanoscale. These guidance documents address defined “engineered nanomaterials” (More et al., 2021a) and food additives containing a fraction of smaller particles (More et al., 2021b). The EFSA guidance recommends a tiered analysis of nanospecific considerations revolving around the behavior of the NM within the gastrointestinal (GI) tract. This includes dissolution dynamics, cellular uptake, transcytosis, and potential disruption of the intestinal barrier. Additionally, genotoxicity and the accumulation potential of the NM are assessed. This tiered analysis offers a unique opportunity to integrate *in vitro* testing into the regulatory process. The guidance document additionally proposed a stepwise approach, focusing on *in vitro* assays related to cytotoxicity (cell death), oxidative stress, (pro) inflammation, and impairment of the intestinal barrier. Furthermore, *in vitro* dissolution testing under simulated lysosomal and GI conditions is recommended to obtain a more exhaustive picture of the complex biological interactions of ingested NMs in the GI tract. In order to develop case studies implementing the two guidance documents, the EFSA launched a pilot project using nanocellulose (NC) as an emerging material in the food sector, and additional case studies are ongoing in the remit of an EFSA-funded project called NAMS4NANO, where various NMs are characterized and tested in different *in vitro* models.

### 1.2 Intestinal barrier disruption, an adverse outcome triggered by food NMs?

The gut barrier plays a crucial role as both a protective barrier that separates the internal blood from the luminal content and a selective barrier that regulates the flux of water, ions, and essential dietary nutrients. The intestinal barrier consists of a multilayer system encompassing a chemical layer containing the antibacterial proteins secreted by Paneth cells, a mucus layer secreted by goblet cells, an epithelial layer, and the cellular immune system. Enterocytes and goblet cells are the most studied cell types. Alteration of one or many of those layers leads to increased intestinal permeability, also called intestinal hyper-permeability or leaky gut syndrome, enhancing translocation of bacteria, bacterial products (such as lipopolysaccharides), and undigested nutrients from the intestinal lumen into the systemic circulation (Kinashi and Hase, 2021). Many diseases arise or are exacerbated by a leaky gut, including diarrhea, inflammatory bowel disease, celiac disease, autoimmune hepatitis, or type 1 diabetes (Aleman et al., 2023; Marasco et al., 2022; Miele et al., 2009; Anand and Mande, 2022). In addition, individuals with a leaky gut are more vulnerable to toxicity driven by chemical exposure as dietary pesticides or food chemicals can more easily enter the systemic blood and reach the target organs (Gama et al., 2022). Although it can be argued that intestinal barrier disruption is an intermediate event toward various clinical outcomes, given its central role, in this study, we advocate considering this event as an adverse outcome when assessing the toxicity of food NMs.

### 1.3 Development of an Adverse Outcome Pathway to support the NM risk assessment in the food sector

Although the underlying mechanisms remain evasive and evidence has not been systematically gathered or produced, a mechanistic-based approach would be highly instrumental in assessing the impact of food NMs on intestinal barrier integrity. Such a risk assessment based on mechanistic reasoning requires relevant adverse outcome pathways (AOPs). The OECD defines the AOP as a description of a logical sequence of causally linked events (key events, KEs) at different levels of biological processes, which follows exposure to a stressor and leads to an adverse outcome (AO) in humans or wildlife (Garcia-Reyero and Murphy, 2018). Hence, a KE describes a measurable and essential change in a biological system that can be quantified in experimental or clinical settings. Then, the strength of the relationship between two KEs (key event relationship, KER) is first established by biological plausibility (e.g., binding to a receptor induces a signaling cascade) and then by evaluating the current evidence supporting this causal link; this approach can also highlight gaps in the knowledge or inconsistencies between studies. Data discrepancies can arise from variations in experimental settings and physicochemical properties of NMs. The development of an AOP is a dynamic process built from hypothesized KEs toward the refinement of KEs and KERs by assembling evidence found in the literature or newly produced (Vinken, 2018). AOP development guidance was established by the OECD (OECD, 2016), and already developed AOPs describing various AOs are stored in the publicly accessible repository platform called AOP-Wiki (https://aopkb.oecd.org/). There is no explicit quality assurance for AOPs in AOP-Wiki, except for those that have undergone the OECD review process (OECD 2018), which makes them compliant AOPs compliant for potential regulatory use.

Initially developed to support new approach methodology (NAM)-based chemical risk assessment, the framework was recognized as instrumental in prioritizing and developing testing strategies for NMs (Halappanavar et al., 2019). Most AOPs developed for chemicals should also be applicable to NMs, considering certain adaptations at the molecular initiating event (MIE) and early KE levels (Clerbaux et al., 2022). For example, AOP 144, leading to liver fibrosis, was proven to be nano-relevant as endocytic lysosomal uptake is described as a nano-relevant MIE connected to lysosomal disruption and mitochondrial dysfunction, leading to cell death in the liver (Gerloff and Landesmann, 2024; Murugadoss et al., 2021; Gerloff et al., 2017). Simple *in vitro* models for testing the potency of NMs were associated with the MIE and early lysosomal and mitochondrial KEs in this AOP (Murugadoss et al., 2021). These early KEs lead to cell death.

Building on “tissue injury,” Halappanavar et al. proposed a methodology for identifying NM-relevant KEs based on the plausibility, measurability, and regulatory importance of the KEs (Halappanavar et al., 2019). The authors identified inflammation (increased pro-inflammatory mediators and leukocyte recruitment/activation), oxidative stress, and cell death as the main upstream KEs to tissue injury. Reported endpoints and associated assays were identified, allowing for a quantifiable measurement of these KEs. Finally, in line with the increasing recognition of the central role of the leak gut, ‘intestinal barrier disruption’ was recently added to AOP-Wiki in the COVID-19 context (Clerbaux et al., 2022). However, although the ingestion of food NMs increases the possibility of an adverse outcome on the intestinal barrier, there are currently no AOPs supporting the toxicity of NMs on the intestinal barrier following their cellular uptake in the intestine.

In this study, we developed an AOP proposing a toxicological pathway toward intestinal barrier disruption, following the intestinal uptake of NMs via lysosomal and mitochondrial dysfunction. This AOP was developed by leveraging information from existing AOPs published in AOP-Wiki and based on biological plausibility. Following the establishment of the AOP, we extracted current evidence from the scientific literature for a targeted selection of NMs highly relevant to the food sector, including ZnO, CuO, FeO, SiO2, and Ag NMs and nanocellulose. They have gained increasing interest due to their potential applications in different areas (biocides or pesticides, cosmetics, nutrient feed and food additives, food supplements, and food contact materials) and have also been extensively investigated in nanotoxicology studies. However, conflicting data on NM oral exposure in humans, uncertainties regarding their fate in the human body, the pristine NM physicochemical changes potentially occurring during the digestive process, and the low human relevance of animal models (e.g., pH in the stomach) still in use highlight the need for further investigation. This also emphasizes the importance of developing integrated approaches to testing and assessment (IATAs) and AOPs, as recommended by the EFSA Roadmap (Escher et al., 2022) With this purpose, our in-depth literature review allowed us to highlight current knowledge gaps and data inconsistencies guiding future research.

## 2 Methods

### 2.1 AOP-Wiki search to identify intestinal AOPs with NMs reported as stressors

In the prototypical stressor section of AOP-Wiki, the keywords “nanoparticles,” “NPs,” “nanosized particles,” “nanomaterials,” and “nanotubes” were used to filter AOPs in AOP-Wiki, which report a nanosized material as a stressor. For each AOP, the AOP number, MIE, AO, and status information have been extracted.

### 2.2 AOP development strategy

**FIGURE 1:**
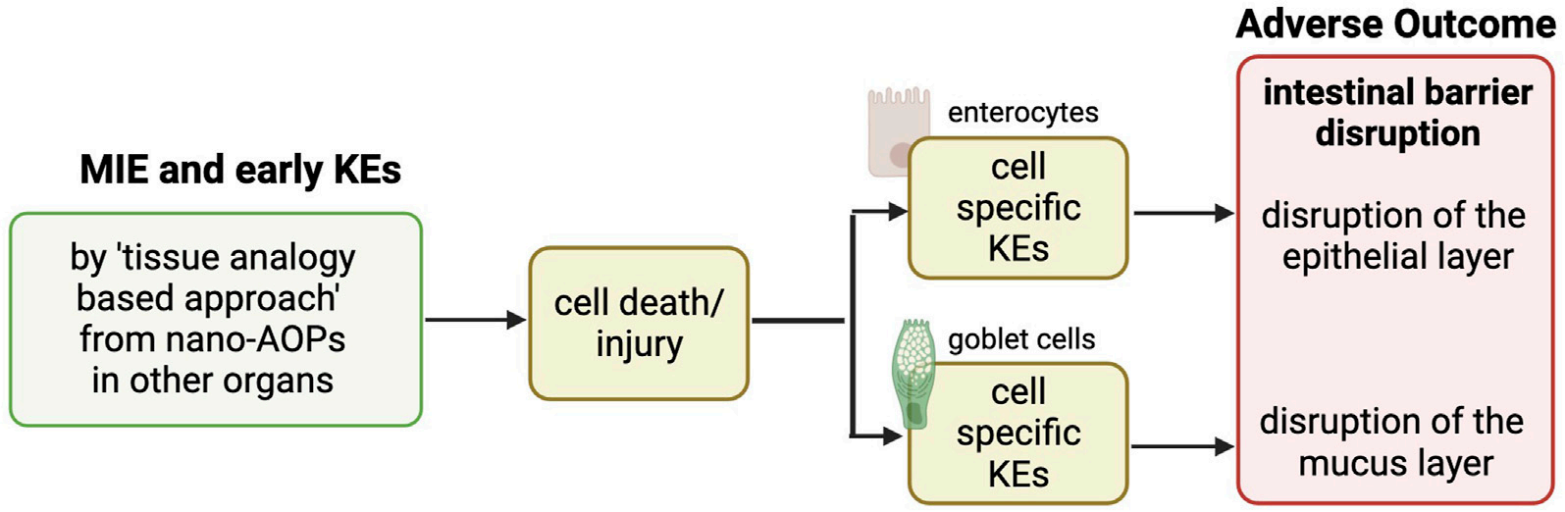
AOP development strategy.

The AOP was first built based on biological plausibility and by leveraging existing KEs in another NM-relevant AOP. We started with the apical outcome, “intestinal barrier disruption,” which we postulated was due to “cell death/injury” of the different intestinal cell types, identified as a main KE upstream of tissue injury in nano-relevant AOPs (Halappanavar et al., 2019), and this rationale is based on the fact that the intestinal barrier is composed of different layers of specific cell types, whose death or injury impairs the barrier function. In this study, we focused on enterocytes and goblet cells. The identification of the intermediate cell-specific KEs between “cell death/injury” and “intestinal barrier disruption” was based on biological plausibility and further refined, following a literature search. On the other side of the pathway, the MIEs and initial KEs defined in the nano-relevant AOP for the liver, considering NM-induced ‘mitochondrial dysfunction’ leading to “cell death/injury,” were extrapolated via a tissue analogy-based approach as upstream KEs (Figure 1).

To verify the relevance of the proposed AOP for the food sector, we focused the literature research on NMs used as food additives and novel foods. Zinc oxide (ZnO) NMs are micronutrient supplements and food fortification ingredients that can release Zn ions intracellularly after being absorbed by enterocytes (Youn and Choi, 2022). Silicon dioxide (SiO_2_) NMs (E551) are food additives that exhibit size-dependent cytotoxicity in *in vitro* studies (Dong et al., 2020). Iron oxide (FeO) NMs (E17) serve as food additives and flavorings (Ansari et al., 2019); their impact on the intestinal barrier is yet to be explored. Nanosilver (Ag NM, E174) is an antimicrobial material used as a colorant or food contact material that most properly exerts its toxicity through silver ion release, but conclusive studies are lacking (Bredeck et al., 2021). Copper oxide (CuO) NMs are used as nutrients, food supplements, and pesticides and share potential ion-driven toxicity mechanisms with ZnO and Ag NMs (European Food Safety Authority EFSA, 2009).

### 2.3 Literature search

The literature search was performed both automatically using AOP helpFinder and manually in PubMed based on selected keywords, followed by a selection procedure of the relevant studies based on predefined exclusion criteria.

#### 2.3.1 Keyword selection and literature screening

The automatic search was performed using the AOP-helpFinder tool (Carvaillo et al., 2019), a hybrid approach that combines text mining procedures and graph theory to automatically identify the literature containing co-occurrence between the stressor(s) and KE(s) of interest. To do so, two lists of keywords have been created based on expert knowledge:

Stressors: Zinc oxide, silicon dioxide, iron oxide, silver, copper oxide, nanopesticide, and nanocellulose.
KEs: Intestinal barrier, gut, enterocytes, intestinal goblet cells, Paneth cells, M cells, intestinal stem cells, Lgr5-positive cells, and intestinal endocrine cells.

The parameters used to screen the abstracts in AOP helpFinder were 1) dismissing the first 20% of the abstract and 2) applying the refinement filter, a second analysis that used a lemmatization process to contextualize words with common stems (e.g., tests, testis, and test). The abstracts were provided as outputs in a table along with PMID identifiers.

Then, the titles and abstracts of publications within the PubMed database (as of March 2024) were screened using the following keywords:

“NM” AND “nano*” AND “gut”
“NM” AND “nano*” AND “intestinal barrier”
“NM” AND “nano*” AND “intestinal toxicity”
“NM” AND “nano*” AND “intestinal cells”
Here, NMs were zinc oxide, silver, silica dioxide, copper oxide, iron oxide, nanocellulose, and nanopesticide.

#### 2.3.2 Exclusion criteria

For both literature searches, we manually excluded abstracts that were duplicates, not in English, not containing primary data (e.g., reviews), not related to food NMs, not related to the intestinal barrier, or related to nanomedical applications. In addition, studies involving human cells and samples and rodent studies were selected, while studies using environmentally relevant species (e.g., worms, flies, Daphnia, and fish) were excluded.

### 2.4 Extraction of relevant information

#### 2.4.1 Collecting evidence

The entire publication of the identified abstracts was read by the authors. First, the origin of the NM was reported, followed by the biological system used in the study (e.g., intestinal human cells or rodent studies). Then, for each KE, the information regarding the measure was reported as equal, increased, or decreased compared to the control (e.g., experimental settings without exposure to the NM). The lowest dose at which a change was observed was retrieved. When no significant differences were observed, the highest dose used in the study was recorded. Only when the duration of exposition was different from 24 hours, the information was reported. In addition, the type of assays used to produce the evidence was retrieved from the studies. Finally, the references of the publication were collected.

#### 2.4.2 Physicochemical score

To evaluate the level of characterization reported by the authors in the studies, we allocated 0 points if no information was mentioned regarding the size, 0.5 point if the size of the pristine material was measured, and 1 point if both pristine and NM sizes in the experimental medium were measured. If another property was described, we allocated one extra point. Finally, if more than one additional property, in addition to size, was measured, we allocated two extra points. These grades were arbitrarily assigned, ranging from the lowest (0) to the highest (Vincentini et al., 2023) based on the number of NM measurements performed. Of note, for compounds from the JRC repository (Rasmussen et al., 2013; Singh et al., 2012), the highest grade of 3 was assigned as they are thoroughly characterized.

## 3 Results

### 3.1 AOPs reporting NMs as stressors in AOP-Wiki

To date 15 AOPs are present in the AOP-Wiki that report nanosized material(s) as a stressor with only AOP173 endorsed by OECD (Table 1), refers to the correlation between the NM cellular uptake by endocytic lysosomal absorption and the consequent mitochondrial dysfunction leading to liver fibrosis (Gerloff and Landesmann, 2024). Other AOPs illustrate the NM-mediated lung toxicity (shown as gray in Table 1). AOP 237, 303, 302, and 319 are under development as part of different OECD projects. Finally, there are several AOPs proposed that are not currently part of the OECD process. Not pertaining to the lungs, AOP 209 connects silica nanoparticle-induced disruption of cholesterol to hepatotoxicity, while AOP 207, AOP 208, and AOP 210 suggest pathways leading to infertility.

**TABLE 1:** AOPs reporting NMs as stressors in AOP-Wiki.

Stressor	AOP ID	MIE	AO	Status
NPs	144	Endocytotic lysosomal uptake	Liver fibrosis	OECD under review (1.47)
Carbon nanotubes and carbon nanofibers	173	Substance interaction with the lung resident cell membrane components	Pulmonary fibrosis	OECD-approved (1.32)
Insoluble nanosized particles	237	Substance interaction with the lung resident cell membrane components	Atherosclerosis	Under development (1.55)
High aspect ratio material	303	Frustrated phagocytosis	Lung cancer	Under development (1.86)
“Wide range of nanomaterials”	302	Inhibition of lung surfactant function	Decreased lung function	Under development (1.87)
Nanoparticles, SARS-CoV-2	319	Induced dysregulation of ACE2	Lung fibrosis	Under development (1.96)
Silica NPs	481	Increased reactive oxygen species	Respiratory dysfunction	Not OECD
Carbon nanotubes	241	Activation, latent transforming growth factor beta 1	Pulmonary fibrosis	Not OECD
Nanomaterials	451	Substance interaction with lung resident cell membrane components	Lung cancer	Not OECD
Carbon nanotubes	409	Frustrated phagocytosis	Increased risk of mesotheliomas	Not OECD
Nanomaterials, SARS-CoV-2	392	Fibrinolysis decrease	Hyperinflammation	Under development (1.96)
Silver NPs	207	Activation; NADPH oxidase	Reproductive failure	Not OECD
Titanium dioxide NPs	208	?	Reproductive failure	Not OECD
Silica NPs	209	?	Hepatotoxicity	Not OECD
Graphene oxide NPs	210	?	Reproductive failure	Not OECD

In addition, outside AOP-Wiki, at least five AOPs identifying NMs as stressors and leading to lung outcomes are described in the literature in humans. Two AOPs starting from an increased substance interaction and leading to lung emphysema and lung fibrosis, respectively, have been proposed (Halappanavar et al., 2020). Literature reports suggesting potential hazards of TiO_2_ served for the development of one putative AOP, leading to lung cancer (Nymark et al., 2021; Braakhuis et al., 2021). An AOP for graphene-family nanomaterial-induced lung damage was developed (Ding et al., 2023), as well as an AOP describing Ag NM toxicity toward the respiratory tract (Nicholas et al., 2021). Regarding intestinal outcomes, a plausible AOP was proposed following the ingestion of TiO_2_ nanoparticles, which could eventually lead to colorectal cancer (Rolo et al., 2022). This AOP is not included in AOP-Wiki. An AOP-oriented study also assessed cytotoxicity, oxidative stress, genotoxicity, perturbation of the cell cycle, and apoptosis in human intestinal cells, following Ag NM exposure, based on KEs present in AOPs reporting Ag NMs as a stressor; however, no intestinal outcomes were reported (Kose et al., 2023).

Thus, although food NMs enter the human body through the oral route, increasing the possibility of adverse effects on the gut barrier, there are currently no AOPs developed supporting the toxicity on the intestinal barrier function, following cellular uptake by intestinal cells of ingested NPs. A structured approach to assess these effects can be instrumental. In this study, we aimed to develop an AOP specifically focused on the impact of NMs on the intestinal barrier. This AOP construction relied on biological plausibility to link NM cellular uptake with the disruption of the intestinal barrier. We then aimed to evaluate this framework by extracting evidence from published research. Finally, our analysis aimed to identify any knowledge gaps or inconsistencies in the evidence supporting this AOP.

### 3.2 Biological plausibility for an AOP linking NM uptake to intestinal barrier disruption

NM-relevant MIEs identified in AOPs in other tissues might be linked to the intestinal tissue. AOP 144 is at the second highest stage of AOP development. The MIE, endocytic lysosomal uptake, leads to lysosomal disruption, which induces mitochondrial dysfunction and leads to cell injury/death. This is of interest because a recently proposed AOP network linked NM-induced mitochondrial dysfunction to existing AOs in the lung, liver, and cardiovascular and nervous systems (Murugadoss et al., 2023). The authors identified that NM-induced mitochondrial toxicity is crucial for many tissues, but interestingly, they did not mention the intestinal epithelium.

**FIGURE 2:**
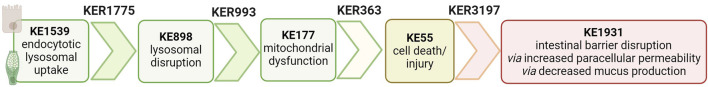
Proposed AOP linking nanomaterial uptake to intestinal barrier disruption based on biological plausibility.

Hence, we propose an AOP that is biologically plausible (Figure 2).

#### 3.2.1 NM endocytosis to intestinal cell death/injury

The biological plausibility that endocytic lysosomal uptake of NMs leads to lysosomal disruption is high and described in KER1775 in AOP-Wiki. In brief, endocytosis, discovered by Christian de Duve, is an active transport in which molecules are transported into the cell by engulfing them in the plasma membrane, which then forms a vesicle containing the ingested material inside the cell. Vesicles rapidly fuse to form larger compartments, known as endosomes. As detailed in KE1539 in AOP-Wiki, there are different assays to evaluate NM cellular uptake (Box 1). Internalized material by endocytosis is then transferred to lysosomes, which are vacuoles containing hydrolytic enzymes in an acid environment, to degrade the ingested material (Villamil Giraldo et al., 2014). Regarding NMs, once they are taken up by a cell and transported to the lysosome, the acidic milieu herein can either enhance their solubility, or they remain in the initial nano-form. Both situations can cause lysosomal swelling, followed by lysosomal disruption and the release of pro-apoptotic proteins (Wang et al., 2013; Cho et al., 2011). Lysosome disruption can be measured as in Box 2 from KE898 in AOP-Wiki.

BOX 1 **How KE1539 can be measured.**

• Transmission electron microscopy (TEM) is appropriate for visualizing NMs inside cells since light microscopy fails to resolve them at a single particle level.
•Confocal laser scanning microscopy (CLSM) combines high-resolution optical imaging with depth selectivity, which allows for optical sectioning.
•Inductively coupled plasma mass spectrometry (ICP-MS) is a type of mass spectrometry capable of detecting metals and several non-metals at very low concentrations.
•Inductively coupled plasma optical emission spectrometry (ICP-OES) enables the detection of the presence of elements based on their emission of light when excited by plasma energy.
•Fluorescence-activated cell sorter (FACS) is a specialized type of flow cytometry for sorting a heterogeneous mixture of biological material based on the specific light scattering and fluorescent characteristics of each cell.
•Atomic absorption spectroscopy (AAS) is a widely used technique for the quantitative determination of elements in a variety of samples by measuring the absorption of specific wavelengths of light by the element of interest.
•EDS is used in conjunction with scanning electron microscopy (SEM) to analyze the elemental composition of materials.

BOX 2 **How KE898 can be measured.**

•LysoTracker Green is regularly used to assess lysosomal acidification.
•Changes in morphology can be observed by using acridine orange, a weak base that accumulates in the acidic compartment of the cell mainly composed of lysosomes. This is followed by flow cytometry, static cytofluorometry, or flow cytofluorometry.
•Lysosomal membrane permeabilization can be visualized by immunostaining lysosomal enzymes such as cathepsin B. More specific staining can be achieved by staining with antibodies against lysosomal membrane proteins.
•Proteomics analysis can offer a comprehensive view of the changes in protein expression or post-translational modifications associated with lysosome disruption. By identifying alterations in protein profiles, proteomics can provide valuable insights into the molecular mechanisms involved in lysosomal dysfunction.
•Confocal microscopy enables high-resolution imaging of cellular structures, including lysosomes. By visualizing lysosomal morphology, localization, and dynamics, confocal microscopy can directly demonstrate alterations in lysosomes caused by disruption. It can also be used in conjunction with specific dyes or probes to track lysosome-related processes in real time.

The biological and causal link between lysosome and mitochondrial toxicity is detailed in KER993. The release of lysosomal proteases due to lysosomal disruption induces mitochondrial dysfunction (KE177), which encompasses a wide variety of changes in the structure and function of the mitochondria. The most reported ones are altered production of ATP, loss of mitochondrial membrane potential (MMP), inhibition of protein complexes in the electron transport chain, and failure to produce enzymes that detoxify ROS (Box 3).

BOX 3 **How KE177 can be measured.**

•Mitochondrial membrane potential (MMP) measurement. JC-1 staining is a common fluorescent dye-based assay used to assess mitochondrial membrane potential, which can be a direct indicator of mitochondrial health and function. In conjunction with fluorescence microscopy or flow cytometry, it is used to quantify changes in mitochondrial membrane potential.
•Enzymatic activity of the electron transport system via the MTT assay is a colorimetric assay where NAD (P) H-dependent cellular oxidoreductase enzymes reflect the number of viable cells.
•ATP content measurement via ATP assay used to signal the presence of metabolically active cells.
•Cellular oxygen consumption: the oxygen consumption rate is an integrative and comprehensive readout of cellular metabolism and mitochondrial function commonly measured using chamber-based platinum electrodes and microplate-based fluorescent readings.
•Proteomic analysis analyzes changes in protein expression levels, post-translational modifications, and interactions within the mitochondria. Proteins commonly associated with mitochondrial dysfunction include cytochrome c and heat shock proteins.

Finally, the biological plausibility of mitochondrial toxicity leading to cell death/injury is well-established in the literature and captured within KER363 included in OECD-endorsed AOP 48 and in AOP 144 under review in AOP-Wiki (Yang and Xiang, 2024). Cytotoxicity (apoptosis/necrosis) can be measured via different assays, as detailed in Box 4 from KE55.

BOX 4 **How KE55 can be measured.**

•WST-1 and MTT are colorimetric assays where NAD (P) H-dependent cellular oxidoreductase enzymes reflect the number of viable cells.
•The ATP assay was used to signal the presence of metabolically active cells.
•LDH leakage assay: lactate dehydrogenase (LDH) is a soluble cytoplasmic enzyme released outside the cell when the plasma membrane is damaged and detected with a tetrazolium salt.
•Propidium iodide (PI) is an intercalant and fluorescent molecule used to stain necrotic cells.
•Neutral red uptake is based on the ability of viable cells to incorporate and bind the supravital dye neutral red in lysosomes.
•Trypan blue assay is used to calculate the cell number and percentage of viable cells in a cell population. It is based on the use of the trypan blue dye that is permeable for dead cells but impermeable for normal cells due to a damaged plasma membrane.
•TUNEL detects DNA fragmentation from apoptotic signaling cascades.
•Caspase activity assays detect and quantify caspase activity within cells, providing valuable insights into the apoptotic process.
•Hoechst/DAPI staining binds specifically to DNA molecules present in the cell nucleus if the cell membrane is not intact and the stain can penetrate. It is a fluorescent dye. When exposed to ultraviolet light, the dye bound to DNA emits blue light, making the nucleus easily visible under a fluorescence microscope.
•Acridine orange visualizes nuclear changes and apoptotic body formation under a fluorescent microscope.
•Annexin V/PI staining, or Annexin V and propidium iodide (PI) labeling, of cells is a technique used to identify cell death and distinguish between its different pathways: apoptosis, programmed cell death, and necrosis.
•Impedance-based cellular assays (IBCAs) allow for the non-invasive and instantaneous detection and monitoring of cell responses to chemical and biological agents. Small changes in the impedance of the current flow in the cell culture substrate allow determining the events of cell adhesion, spreading, growth, motility, and death.
•Live/dead staining is a mixture of two fluorescent dyes that differentially label live and dead cells.
•Hematoxylin and eosin (HE) staining of tissue sections: the nuclei are stained purple, while the cytoplasmic components are pink.

BOX 5 **How KE1931 can be measured.**

Paracellular permeability
  •Transepithelial electrical resistance (TEER) measures the barrier integrity of the cell layer.
  •Monitoring the passage of fluorescent molecules (FITC-dextran, sodium fluorescein, or Luciferase yellow (LY) provides the apparent permeability coefficient (Papp) using the formula Papp= (dQ/dt)/AC0, where dQ/dt is the transport drug/NM per unit time (mg/s); A is the area of the transport membrane (cm^2^); and C0 is the initial concentration of sample solutions (µg/mL).
  •Staining of tight junctions (TJ), such as ZO-1 and occludin, by immunofluorescence.
Mucus secretion and thickness.
  •Alcian blue staining can visualize the mucus layer, structure, and thickness.
  •Histology staining (HE) was used to assess intestinal mucosal thickness and the number of goblet cells.

#### 3.2.2 Intestinal cell death leads to intestinal barrier disruption

The biological plausibility that damaged/dying enterocytes or goblet cells lead to an increase in paracellular permeability due to disruption of the epithelial monolayer and a decrease in mucus secretion and thickness, respectively, is high, but the causal link is not captured in AOP-Wiki. We created KER3197 in the AOP-Wiki linking cell death/injury to intestinal barrier disruption. The intestinal barrier is a multilayer system composed of a chemical layer containing the antibacterial proteins secreted by Paneth cells, a mucus layer secreted by goblet cells, a one-cell-thick epithelial layer attached together through tight junction (TJ) proteins, and a cellular immune layer. Intestinal permeability describes the movement of molecules from the lumen to the blood, making it a measurable feature of the intestinal barrier function. Transcellular permeability encompasses passive diffusion from the apical to the basal side (lumen to blood), vesicle-mediated transcytosis, and membrane receptor-mediated uptake. Paracellular permeability is regulated by the tight junctions between adjacent cells and by the integrity of the epithelium. The disruption of the intestinal barrier (KE 1931) can be caused by damaging one or many layers due to cell death/injury of the specific associated cells. In this study, we will focus on enterocytes and goblet cells and the associated alteration of epithelial monolayer integrity and the mucus layer, respectively. Paracellular permeability and mucus secretion/thickness can be measured as described in Box 5.

### 3.3 Evidence assessment

#### 3.3.1 Selection of relevant publications

**FIGURE 3:**
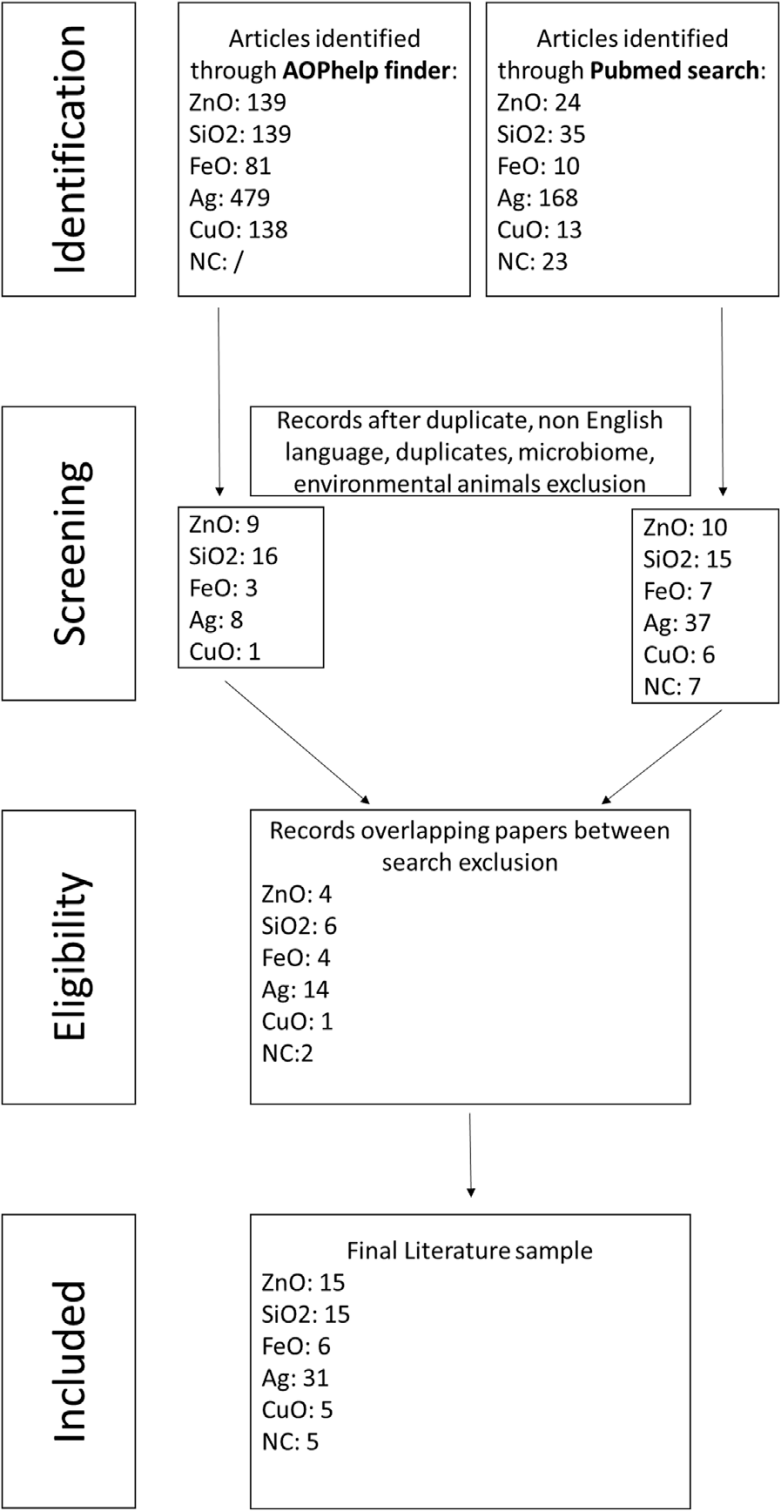
Selection process to identify the relevant publications.

Based on the selected keywords, we obtained 1,249 papers from the combined PubMed database and AOP helpFinder search. We excluded 140 for ZnO, 137 for SiO_2_, 77 for FeO, 588 for Ag, 143 for CuO, and 14 for NC papers due to replicates or overlaps between the two search methods, non-English language, or because the studies did not focus on NM effects on the intestinal barrier in humans or rodents (Figure 3). For nanopesticides, no results were obtained from either search.

#### 3.3.2 Empirical support for KERs

For each selected publication, we reported the stressor used in the study along with a physicochemical (PC) score. In their guidance, EFSA insists on the importance of precise physicochemical characterization of NMs by reporting composition, size, size distribution, shape, charge, agglomeration state, and surface composition. The level of NM characterization in the studies was reported as grades ranging from the lowest (0) to the highest (Vincentini et al., 2023) number of physicochemical properties measured. Then, we reported the biological system used, including the cell type, whether it was proliferating (P) or differentiated (D), cultured in transwell (T) or glass side (G), and whether the treatment was applied to the apical (AP) or basolateral (BL) side. For rodent studies, the route of exposure, duration, and dose were retrieved. Then, the measure regarding each KE was reported as equal (=), increased (+), or decreased (−) compared to the control. For example, if transepithelial electrical resistance (TEER) values decreased upon exposure to NM, this means that the intestinal barrier disruption increased (+). The dose indicated is the lowest that induced a change (+/−) in the study and the highest dose used when no changes were reported (=). The types of assays used to create evidence were retrieved from the studies and are shown in Tables 2–7).

**TABLE 2:** Evidence from the published literature supporting AOP 530 for ZnO NMs.

NM	PC	System	KE1539 Endocytotic lysosomal uptake		KE898 Lysosomal disruption		KE177 Mitochondrial dysfunction		KE55 Cell death/injury		KE1931 Intestinal barrier disruption		Reference
			+/−/ =	Assay	+/−/ =	Assay	+/−/ =	Assay	+/−/ =	Assay	+/−/ =	Assay	
ZnONP (544906, <50 um 67745, <100 um, Sigma-Aldrich)	3	Caco-2 DT	+	ICP-MS (614 uM)			=	MTT	=	MTT	= =	TEER Papp (614 uM)	Mittag et al. (2022a)
		HT29-MTX DT	+	ICP-MS (614 uM)			=	MTT	=	MTT Alcian blue staining	- =	TEER Papp (307 uM)	
ZnONP (544906 Sigma-Aldrich)	3	Caco-2 DT									+	TEER (100 ug/mL)	Colombo et al. (2019)
ZnONP (<100 nm Sigma-Aldrich)	3	Caco-2 P P	+	ICP-AES									Jeon et al. (2020)
Digested ZnONP (544906, <50 um 67745, <100 um Sigma-Aldrich)	3	Caco-2 DT	+	ICP-MS (307 uM)			=	MMP	=	MTT TEM	=	TEER Papp (614 uM)	Mittag et al. (2022b)
		Caco-2 + HT-29 DT	+	ICP-MS (307 uM)			=	MMP	=	MTT TEM	=	TEER Papp (614 uM)	
Intragastric ZnONP (<50 nm Sigma-Aldrich)	3	30 days, mouse							+	H&E	+	Histology (mucosa) RT qPCR (TJ)	Zhang et al. (2020)
Digested ZnONP (<100 nm)	0.5	Caco-2 D					+	TEM	+	MTT, LDH 0.523 uM			Berni Canani et al. (2010)
Digested ZnONP	3	Caco-2 P, DT							-	Calcein AM/PI (0.097 mg/mL)	-	TEER IF (TJ)	Moreno-Olivas et al. (2019)
Digested ZnO NP (nanoscale material <10 nm)	0.5	Caco-2 PP					+	WST-1 (20 ug/cm^2^	+	LDH WST-1			Gerloff et al. (2009)
ZnO NP (nanoscale material <10 nm)	3	Caco-2 D	+	ICP-OES			+	WST-1 (20 ug/cm^2^	+	WST-1			Gerloff et al. (2013)
NM110	3	Caco-2 transwell/gut-on-chip					+	WST-1 (50 ug/mL)	+	WST-1 (50 ug/mL)			Kulthong et al. (2021)
ZnO NPs (Sumitomo)	3	Caco-2 PP	+	ICP-AES			+	WST-1 (10 ug/mL)	+	WST-1 (10 ug/mL)			Go et al. (2018)
ZnO + vit C + CPP	3	GES-1					+	MMP	+	LDH			Gu et al. (2018)
ZnO NPs (American Elements)	2.5	INT-407 proliferating	+	ICP-AES					+	LDH (50 ug/mL)			Kim et al. (2017)
ZnO NPs (Aladdin 30 nm)	0.5	*In vivo* mouse colon									+	Histology (mucosa) RT qPCR (TJ)	Yan et al. (2022)
ZnO/Zn^2+^ (Merck Corp)	3	In food, 270 d mouse jejunum	=	TEM	=	TEM			=	Histology	+	Histology (mucosa) RT qPCR (TJ)	Liu et al. (2017)

**TABLE 3:** Evidence from the published literature supporting AOP 530 for CuO NMs.

NM	PC	System	KE1539 Endocytotic lysosomal uptake		KE898 Lysosomal disruption		KE177 Mitochondrial dysfunction		KE55 Cell death/injury		KE1931 Intestinal barrier disruption		Reference
			+/−/ =	Assay	+/−/ =	Assay	+/−/ =	Assay	+/−/ =	Assay	+/−/ =	Assay	
CuO NM (10 nm, PlasmaChem, GmbH)	3	Caco2/RajiB TD AP	+	ICP-OES					=	SEM IF nuclei Romanowsky staining	+ + +	TEER IF (ZO1) Papp	Ude et al. (2019)
		Caco-2/RajiB-MTX TD AP											
CuO commercial (Sigma),ethanol, and water	2	Caco-2 TD AP or BL									+	TEER CuOc (10 μg/mL, BL) CuOe and CuOw (100 μg/mL, AP)	Bertero et al. (2021)
CuO (<50 nm, Sigma)	3	EpiIntestinal™ (SMI-100)					+	MTT	+	MTT (40 μg/mL)			Henson et al. (2019)
		Rat IEC-6					+	MitoTracker (0.08 μg/mL)	+	MTS 4 μg/mL			
CuO (10 nm PlasmaChem, GmBH, Berlin)	3	Caco-2 PL, TD AP	+	ICP-OES			+	Alamar blue (2.44 μg/cm^2^)	+	Alamar blue (2.44 μg/cm^2^)	+ + +	TEER IF (ZO1) IF (DAPI)	Ude et al. (2017)
CuO in lettuce	0.5	Caco-2 TD, AP 2 h	+	TEM	+	TEM	+	TEM	+ +	LDH MTT			Li et al. (2020)

**TABLE 4:** Evidence from the published literature supporting AOP 530 for FeO NMs.

NM	PC	System	KE1539 Endocytotic lysosomal uptake		KE898 Lysosomal disruption		KE177 Mitochondrial dysfunction		KE55 Cell death/injury		KE1931 Intestinal barrier disruption		Reference
			+/−/ =	Assay	+/−/ =	Assay	+/−/ =	Assay	+/−/ =	Assay	+/−/ =	Assay	
VENGES Fe_2_O_3_ Digested (300 nm)	3	Caco-2, HT29 RajiB TD AP	+	ICP-MS 2/4 h					=	Live/dead LDH	=	TEER	DeLoid et al. (2017)
PLGA-PEG Fe_3_O_4_ Digested (100 nm, Colorobbia Consulting, Italy)	3	Caco-2 PT/PL					+	WST-1 50 μg/mL	+	WST-1 50 μg/mL			Antonello et al. (2022)
		HCT116					=	WST-1	=	WST-1			
		HCoEpiC					+	WST-1 20 μg/m	+	WST-1 20 μg/mL			
		Caco-2 TD AP	=	NTA Ferrozine			=	WST-1	=	WST-1	= =	TEER LY	
											-	qRT-PCR (TJP1, OCLN, and CDN5)	
E172 Two yellow FeO(OH), two red Fe_2_O_3_, one orange Fe_2_O_3_+FeO(OH), and two Black Fe_3_O_4_ (<170 nm) Digested	3	Caco-2 PT/PL					=	MTT 100 μg/mL	= =	MTT 100 μg/mL IBCA Red 1 and orange 2			Sieg et al. (2024)
									+	IBCA Yellow 1/2 Black 1/2 >5 μg Fe/mL			
		Caco-2 TD, AP	+	AAS					=	IBCA			
Amino PVA USPIO (polyvinyialchol/polyvinylamine amino) (40 nm)	3	Caco-2 and HT29 spheroids	+	Prussian blue, nuclear red									Kenzaoui et al. (2012)
		Caco-2 HT29 PL/PT	+ -	Prussian blue, nuclear red TEM (21 μg/mL)			=	MTT	=	MTT			
		Caco-2 T/D									=	LY	
		Caco-2/HT29 T/D - AP									=	LY	
PAA-coated Fe_3_O_4_ NM (200 nm, Chemichell)	3	Caco-2 T/D	+	AAS TEM IBM 14 μg/mL					=	CellTiter-Blue			Lichtenstein et al. (2017)
		Caco-2/HT29/RajiB T/D - AP	+	AAS							=	TEER Papp 200 μg/mL	
FeO digested (698 nm, China n/a)	2	Mice, 150 mg/kg/d 30 days Intragastric							+	H&E			Li et al. (2023)
		Caco-2 T/D 100 μg/mL AP									+	Papp 100 μg/mL	

**TABLE 5:** Evidence from the published literature supporting AOP 530 for SiO_2_ NMs.

NM	PC	System	KE1539 Endocytotic lysosomal uptake		KE898 Lysosomal disruption		KE177 Mitochondrial dysfunction		KE55 Cell death/injury		KE1931 Intestinal barrier disruption		Reference
			+/−/ =	Assay	+/−/ =	Assay	+/−/ =	Assay	+/−/ =	Assay	+/−/ =	Assay	
Sicastarred (micromod)	3	ISO-HAS-1/Caco-2 DT									=	TEER NaFlu	Kasper et al. (2019)
SiO_2_ nanobeads (HiQ-Nano srl)	3	Caco-2-HT29MTX-Raji DT	+	CLSM							+	TEER IF (TJ) 100 μg/mL	Vincentini et al. (2022)
Precipitated and fumed SiO_2_	3	Caco-2 PT-D Caco-2/Raji B-T PL-AP	+	CPE 2 h							+	Papp 6 h	Yoo et al. (2021)
SiO_2_	3	C57BL/6J mice, 3 g/kg/day, 28 days							+ + +	H&E AB-PAS IHC	+	IF TJ	Diao et al. (2021)
Kaolinite (Argiletz, France)	0	Male Wistar rats, 3.4 g/day in food 28 days	+	ESEM/EDX					=	LM TEM SEM	=	Proteomic	Reichardt et al. (2009)
Kaolinite (Argiletz, France)	0	Male Wistar rats	=	TEM					=	TEM			Reichardt et al. (2012)
SAS Food-grade SiO_2_ nanoparticles (E551) dissolution study 24 h	2.5	Caco-2 BBe1, DG, and AP									+	SEM 1 ug/mL 24 h	Yang et al. (2015)
SAS D90 (DMSNs, spherical, 90 and 130 nm)	3	Caco-2/HT29-MTX-E12 PT, D	+	IF					=	AFM IF	=	TEER IF	Iriarte-Mesa et al. (2023)
SAS (10–200 nm)	3	Caco-2 Caco-2/HT29-MTX TD-PT AP, BL	+	CLSM					=	MTT LDH ROS	+	TEER CLSM Papp ZO-1 (NP30)	Cornu et al. (2020)
SAS	3	DLD-1 SW480 NCM 460 PL, PT	+	CLSM					=	MTT, Annexin V/PI ROS	=	TEER CLSM	Setyawati et al. (2015)
SiO_2_ FG-NP, FG-MP, and NFG-NP	2.5	Caco-2 PL, PT							=	MTT, LC20 (62.8 ppm)	+	GSEA	Xu et al. (2022)
SiO2 E 551	2.5	HT29-MTX-E12 PL, PT	+	FACS IF					=	MTS FACS SCGE			Franz et al. (2020)
SAS	3	Male Sprague–Dawley rats	+	HP					=	SCGE HP MDA			Tarantini et al. (2015)

**TABLE 6:** Evidence from the published literature supporting AOP 530 for AgNMs.

NM/provider/size pristine/digestion	PC	Cell model	KE1539 Endocytotic lysosomal uptake		KE898 Lysosomal disruption		KE177 Mitochondrial dysfunction		KE55 Cell death/injury		KE1931 Intestinal barrier disruption		Reference
			+/−/ =	Assay	+/−/ =	Assay	+/−/ =	Assay	+/−/ =	Assay	+/−/ =	Assay	
NM300K (Fraunhofer IME, Germany) 7.74 ± 2.48 nm TEM	3	Caco-2 DT PL AP	+	ICPMS TEM					+	Comet assays 50 μg/mL	=	TEER Papp 50 μg/mL qRT-PCR (TJ)	Vila et al. (2018)
PAA-coated Ag	2	Caco-2, HT29-MTX-E12 Raji-B DT, AP	+	AAS TEM SEM					=	CellTiter-Blue 20 μg/mL	=	TEER Papp 20 μg/mL	Lamas et al. (2024)
AgNP 4.84 ± 2.1 nm	2.5	Caco-2 HT29 DT, AP	+	CLSM							+	TEER Papp RTq-PCR (TJ) 100 μg/mL	Saez-Tenorio et al. (2019)
Ag NPs and AgNO_3_ 20–200 nm	2	Caco-2/TC7 HT29-MTX DT PL AP	+	CLSM					=	Alamar blue 100 μg/mL			Georgantzopoulou et al. (2016)
Ag NP 35 nm	2	C3a	+	CLSM					+	LDH IC50 50 μg/mL			Gaiser et al. (2012)
		Caco-2 DT PL AP											
AgNP 20, 34, 61, and 113 nm	3	Caco-2-RajiB DT, AP									=	TEER Papp 25 μg/mL	Bouwmeester et al. (2011)
AgPURE Rent a Scientist GmbH, TEM 7.02 ± 0.68 nm/Y	3	Caco-2 DT, PL							+	CellTiter-Blue DAPI 15 μg/mL	+	Electrodes	Böhmert et al. (2014)
Ag from mussels 23 nm	0	Caco-2 DT, AP	+	TEM							=	TEER LY 0.0406–0.265 μg/g	Taboada-López et al. (2021)
NM300K 20 nm	1.5	Caco-2 DT, AP							=	LDH 15 μg/mL (3 h)			Polet et al. (2020)
NM300 Ras GmbH, 20 nm	2	Caco-2 HT29MTX DT, AP	+	ICP MS 40 ug/mL									Ricotti et al. (2016)
Poly (acrylic acid)-coated AgNM 3.2 ± 0.1 nm/Y	2	Caco-2 DT AP	+	AAS					+	CellTiter-Blue 40 ug/mL	=	TEER Papp 20–100 μg/mL	Lichtenstein et al. (2015)
AgPURE	3	Caco-2, D, PT	+	AAS	=	Proteomics	+	Proteomic	+	NRU, MTT, CTB, DAPI	=	Proteomic TEER	Braeuning et al. (2018)
AgNO_3_			+	AAS	=	Proteomics	+	Proteomic	+	NRU, MTT, CTB, DAPI	=	Proteomic TEER	
AgNO_3_	2.5	Caco-2, PL, PT	+	TEM-EDX, ICP-MS	+	CLSM	+	ROS 5 μg/mL IPA	+	Cell counting Comet assay 5 μg/mL			Domenech et al. (2021)
AgNM	3	Caco-2, PL, PT	+	TEM-EDX ICP-MS	+	CLSM	+	ROS IPA	+	Comet assay			
AgNM	3	Caco-2 D PT	+	CLSM TEM					=	LDH			Gaiser et al. (2009)
Micro-Ag	2.5	Caco-2 DT AP	+	CLSM TEM					=	LDH			
Ag-PVP (Polyvinylpyrrolidone-capped Silver)	3	Caco-2 HT29-MTX-E12 THP-1, PL, PT T, AP BL	+	SEM			+	ROS	+	LDH WST-1 Comet assay	=	TEER	Kämpfer et al. (2021)
Ag-NPs	2.5	Caco-2, SW480 PL, and PT	+	TEM			=	ROS (DHE)	+	MTT (100 mg/L)			Abbott Chalew and Schwab (2013)
Ag-NPs	2.5	NCM460HCT116 PT, PL	+	TEM	=	Protein	+	ROS WB	+	MTT WB LDH RT-qPCR	=	Microscopy	Jia et al. (2020)
(Polyethyleneimine) PEI-AgNP 4 nm AgNP 19 nm AgNP	3 2.5	Caco-2 PL and PT	+	TEM FTIR			+	ROS NO	+	MTT Annexin V/PI (4.5 μg/mL), LDH			Rufino et al. (2021)
Ag nanoparticles Digested NP	2.5	Caco-2 PL and PT	+	TEM EDS					+	MTT and TEM			Mao et al. (2016)
Pristine AgNPs, Digested AgNPs, and silver nitrate (AgNO_3_)	3/3	Caco-2/HT29-MTX D, PT AP, and BL	+	CLSM spICP-MS	=	CSLM			+	WST-1 CLSM ICP-MS, spICP-MS	=	TEER LY FITC-D	Abdelkhaliq et al. (2020)
Silver nanoparticles (AgPURE)	2.5	Caco-2, PL, PT, and D	+	TEM-EDX			+	Proteomic	=	CellTiter-Blue	+	qRT-PCR	Juling et al. (2017)
Peptide-coated AgNM	2	Caco-2, PL, PT, and D	+	TEM-EDX			+	ROS (DCFH-DA)	+	CTB Annexin-V/7AAD LDH DAPI	+	IBCA	Böhmert et al. (2012)
AgNM Ag-AuNM	2.5	HuTu-80, PL, and PT	+	TEM			+	ROS	+	IBCA			Botha et al. (2019)
AgNM AgNO3	3	Caco-2 THP-1 D, T AP, BL	+	TEM					+	LDH DAPI	+	TEER	Kämpfer et al. (2020)
AgNM+/-coatings	2.5	Caco-2, PL, and PT	+	TEM			+	ROS	+	LDH Annexin- V FITC/PI DAPI			Chen et al. (2016)
AgNM	2.5	Caco-2, D, T, AP, and BL	+	TEM ICP-MS					+	TEER LDH	+	TEER	Mao et al. (2016)
AgNM	2	Caco-2, PL, and PT	+	TEM ICP-MS	+	MN Acridine orange			+	Trypan blue Alamar blue			(Sahu et al.)
AgNM (30 nm) digested	3	Caco-2 PL, P	+	TEM ICP-MS	+	Proteomic	+	Proteomic	+	Proteomic	+	Proteomic	Gioria et al. (2018)
AgNM (20 and 50 nm)	2/1.5	Caco-2 PL, PT	+	TEM, ICP-MS	+	FCM			+	Alamar blue and Trypan blue			Sahu et al. (2016a)
AgNM (20 and 50 nm)	2/1.5	Caco-2 PL, PT	+	TEM, ICP-MS	+	CBM			+	Trypan blue			Sahu et al. (2016b)
AgNM (14 nm)	3	Caco-2, D, and PT	+	TEM and ICP-MS	+	2-DE and MALDI-TOF MS	+	Proteomics IPA	+	CTB DAPI			Oberemm et al. (2016)

**TABLE 7:** Evidence from the published literature supporting AOP 530 for nanocellulose.

NM	PC	Cell model	KE1539 Endocytotic lysosomal uptake		KE898 Lysosomal disruption		KE177 Mitochondrial dysfunction		KE55 Cell death/injury		KE1931 Intestinal barrier disruption		Reference
			+/−/ =	Assay	+/−/ =	Assay	+/−/ =	Assay	+/−/ =	Assay	+/−/ =	Assay	
CNC-140 × 20 nm; CNC-250 × 25 nm; CNC-700 × 25 nm; PCNC-540 × 35 nm; CNF-50 nm CNF-80 nm TCNF-250 × 25 nm synthetized	3	Caco-2 TD					+	MTS CNC-250 CNF-80 (50 μg/mL)	+	LDH CNC-250 (50 μg/mL)	= =	Papp IF TJ(ZO1) CNF-50 nm CNF-80 nm	Mortensen et al. (2022)
CNF/TiO_2_ (Nanostructured & Amorphous Materials Inc.)	2	Caco-2, PL, PT					=	MTT 1000 μg/mL	=	MTT 1000 μg/mL			Yu et al. (2020)
CNF (from wood pulp, University of Maine, 28 nm)	0.5	Caco-2, FHC PL, PT					=	MTT WST-8 1000 μg/mL	=	MTT WST-8 1000 μg/mL			Yu et al. (2019)
CNC (University of Maine, 5–20 nm width, 150–200 nm length)	1	Caco-2 PT, D					=	MTT 10 mg/mL	=	MTT 10 mg/mL	=	Papp	Lin et al. (2021)
CNC type I and II (obtained from microcrystalline NC, Sigma, 5–10 nm width, 200–300 nm length)	3	Caco-2/TC7 PL and PD					=	MTT 5 ng/μL	=	MTT 5 ng/μL			González-Domínguez et al. (2019)

MIE. Consistent data for ZnO, CuO, FeO, SiO_2_, and Ag NMs are recorded for *endocytic lysosomal uptake* using different imaging techniques (i.e., CLSM, SEM, and TEM) and ICP-MS, -OES, or AES for visualization and quantification, respectively (Tables 2–6). NC internalization has not been investigated so far (Table 7), probably because of its challenging organic carbon-based nature that requires specific immunofluorescence staining for detection (Vincentini et al., 2023). In a previous study, we stained CNC, NFC, and BNC by calcofluor and CBM-GFP, and the presence of NM inside the cells was analyzed by measuring the emitted fluorescence by CLSM.

KER1775 and KER993. Very few studies evaluated *lysosomal disruption* after NM uptake in the gut. They are primarily related to Ag NMs assessed using proteomics techniques (Table 6) without being confirmed with other specific assays such as lysosomal staining or specific lysosomal disruption assays. Hence, the weight of evidence for KER1775 is low. Similarly, as lysosomal disruption is almost never assessed, evidence linking it to mitochondrial disruption is almost inexistent; hence, empirical evidence is low for KER993.

KER363. There is limited evidence supporting mitochondrial disruption obtained by no specific assays across CuO, SiO_2_, and NC studies (Tables 3, 5, 7). The cell death/injury was investigated via several assays such as MTT, LDH release, CellTiter-Blue, and DAPI staining *in vitro* after NM exposure. Studies using ZnO NMs indicate a causal relationship between mitochondrial dysfunction and cell death, as assessed by MTT, MMP, WST-1, or TEM (Table 2). However, six studies reported both mitochondrial dysfunction and cytotoxicity, while four other studies using the same ZnO NMs showed consistent cellular uptake without mitochondrial and cellular damage (Table 2) (Mittag et al., 2022a; Colombo et al., 2019; Jeon et al., 2020; Mittag et al., 2022b; Zhang et al., 2020) Several studies indicated a cytotoxic effect of CuO NMs (Table 3). Although assessed in more than half of the selected studies, minor or no significant cell deaths or injuries were reported for SiO_2_ (Table 5) (Cornu et al., 2020; Setyawati et al., 2015); however, none of the studies evaluated mitochondrial damage (Table5). Regarding Ag NMs, several reports indicated changes in mitochondrial membrane potential (Table 6). In particular, smaller silver nanoparticles with higher surface activity led to notable mitochondrial changes and increased oxidative stress (Gaiser et al., 2009; Jia et al., 2020). Similar observations are reported for cell death, which is influenced by the size, coating, and surface charge of Ag NMs (Abbott Chalew and Schwab, 2013; Mao et al., 2016) (Table 6). Regarding NC, only one publication reported both mitochondrial and cytotoxicity dysfunction of the differentiated Caco-2 monolayer after crystal nanocellulose (CNC) and fibrillar nanocellulose (FNC) exposure (Mortensen et al., 2022). Interestingly, among the four types of CNC tested with several nano-scale dimensions, only the one with the medium size induced a decrease in mitochondrial functionality and cell viability. Moreover, among the three types of FNC tested, the one with an intermediate dimension (80 nm) caused mitochondrial dysfunction. Based on these data, empirical evidence for KER363 is considered moderate for food NMs.

KER3197. Temporal and dose–response evidence to support that enterocyte or goblet cell death/injury disrupts intestinal barrier function is limited with some inconsistencies across studies. For ZnO NMs, seven studies investigated both cell injury and the intestinal barrier. Of these, four studies using the same NMs observed no changes in both events *in vitro*, while another *in vitro* study found that digested ZnO NMs increased the intestinal barrier. One mouse study noted that gut mucosa was disrupted following the intragastric administration of ZnO NMs, with decreased *Cldn3* mRNA expression, suggesting impairment of intestinal barrier integrity; another mouse study further supported damage to the intestinal barrier but without histological cell damage (Zhang et al., 2020). Regarding CuO NMs, Ude et al. observed uptake, cell death, and barrier disruption, supporting that CuO NM might trigger differentiated Caco-2 to death-induced barrier disruption (Table 3) (Ude et al., 2017). In a subsequent study, they confirmed the outcomes of Caco2/RajiB and Caco2/RajiB-MTX barrier *in vitro* models using the same type of CuO NMs (Ude et al., 2019). Furthermore, morphological changes in microvilli were also reported by SEM images, supporting the results related to barrier disruption. Interestingly, Li et al. showed that differentiated Caco-2 taking up CuO NM possessed morphological ultrastructure changes (Li et al., 2020). The effect on the organelle’s morphology could be related to cell death. The increased number of vacuoles observed in mitochondria within the cells may be attributed to the escape of NMs from the endocytic pathway. Bypassing lysosomal degradation, these NMs are released into the cytoplasm, leading to impairments in the organelle structure and function (Behzadi et al., 2017). Regarding FeO NMs, various reports showed cytotoxic effects without causing intestinal barrier dysfunction *in vitro* (Table 4). Long-term oral administration of nano-iron oxide in mice caused intestinal damage with loss of villi structures and hepatic dysfunction (Saez-Tenorio et al., 2019). As shown in Table 5, upon ingestion of silica NMs, one mouse study observed cell and intestinal damage, while another study did not observe any changes in rats fed with kaolinite (Diao et al., 2021; Reichardt et al., 2009). Moreover, some *in vitro* studies also reported no cell death or intestinal damage, while two others pointed toward intestinal barrier disruption without evidence of cellular damage (Vincentini et al., 2022; Yoo et al., 2021; Yang et al., 2015). The Ag NM literature is the most prolific in assessing cell and intestinal damage, with 24 studies supporting cell death/injuries and 6 showing no cytotoxicity (Table 6). A causal relationship toward intestinal barrier disruption is supported by four studies, while six others observed cell death without gut barrier disruption. Regarding NC, no negative effect on the intestinal epithelium has been reported in the majority of the studies (Table 7). Hence, empirical support is proposed as moderate for KER3197.

AO. The intestinal barrier disruption is commonly monitored by assessing TEER and TJ expression profile (qRT-PCR, IF) or by measuring the apparent permeability coefficient (Papp) of the membrane, generally with Luciferase yellow or FITC-dextran assays. Although intestinal barrier disruption is partially reported across the studies, the results are heterogeneous in some cases. Barrier disruption was observed *in vitro* and *in vivo* upon SiO_2_ NM exposure but not consistently (Table 5). *In vitro* exposure to Ag NMs led to decreased TEER values, changes in gene expression of TJs, and cellular impedance, supporting impairment of the barrier integrity (Saez-Tenorio et al., 2019; Böhmert et al., 2014; Mao et al., 2016; Juling et al., 2017; Böhmert et al., 2012; Kämpfer et al., 2020; Gioria et al., 2018). Moreover, contradictory Ag NM dose–response data have been observed with both low and high doses causing barrier impairment (Böhmert et al., 2014; Abbott Chalew and Schwab, 2013). Importantly, this variability may depend on the experimental conditions, both the *in vitro* system and nanoparticles used in the studies. For instance, the intestinal barrier disruption assessment by TEER was reported only in some of the studies, whereas others based their results on the indirect observation of cytotoxicity and other criteria such as genotoxicity and proteomics (Sahu et al., 2016a; Gioria et al., 2018; Sahu et al., 2016b; Oberemm et al., 2016). Finally, no significant barrier disruption in NC studies has been observed so far (Table 7).

AOP 53 is currently under development on AOP-Wiki, given its mostly qualitative nature, as empirical support for the dose concordance is not well-established for all of the KERs in the pathway. Additional studies are needed to support the essentiality of the KES and provide evidence on temporal and dose–response relationships for each KER. Based on this data extraction, we summarized the upstream and downstream KEs and weight-of-evidence (WoE) evaluation of KERs for NMs relevant for the food sector, as shown in Table 8.

**TABLE 8:** Summary of weight-of-evidence (WoE) evaluation of KERs in the AOP.

KER ID	Upstream KE	Downstream KE	Biological plausibility	Empirical evidence	Overall WoE	Quantitative understanding
1775	Endocytic lysosomal uptake	Lysosomal disruption	High	Low	Low	Low
993	Lysosomal disruption	Mitochondrial dysfunction	High	Low	Low	Low
363	Mitochondrial dysfunction	Cell death/injury	High	Moderate	Moderate	Low
3197	Cell death/injury	Intestinal barrier disruption	High	Moderate	Moderate	Low

### 3.4 Uncertainties, inconsistencies, and critical gaps

The abovementioned table highlights evidence gaps, particularly for KER1775 and KER993, where lysosomal disruption was almost never evaluated (Table 8). Furthermore, our quantitative understanding is currently limited. In addition, data were not consistent among studies that used various stressors, biological systems, and E-related assays.

#### 3.4.1 Stressors

In general, establishing a clear link between the physicochemical properties of NMs and their uptake into cells is challenging due to the complex interplay of properties. Accurately measuring the physicochemical properties of NMs in the *in vitro* setting is crucial. In this study, we proposed a physchem score to indicate the level of detail in NM characterization in each study. This can lead to conflicting results about the link between material properties and cellular uptake. NMs can enter cells through various mechanisms, like endocytosis or passive diffusion. The dominant pathway can depend on the specific NM characteristics, adding another layer of complexity. In the majority of the studies, the physchem characterization of the selected NMs was assessed, taking into consideration these parameters. However, the variability in both NM properties and assay methodologies poses significant challenges for comprehensive study comparisons. Finally, the dynamic extracellular environment of the gut significantly influences NM interactions with the cell membrane and subsequent uptake. Our literature research confirmed that although reporting physchem characterization of NMs has become more common in recent studies, the minimum essential information, such as NM characterization in digestive fluids or cellular media, is still not consistently reported using standardized methods. This hinders the establishment of reliable causal relationships between physchem properties and biological effects. Several studies suggest that the physicochemical characteristics of the NM, such as size, surface charge, and coating materials, may play a role in promoting or hampering the cellular endocytic uptake (Braeuning et al., 2018; Kämpfer et al., 2021). For instance, smaller size and surface modifications enhance silica NM uptake, as shown by an increased uptake of smaller particles (30–130 nm) compared to larger particles (200 nm) (Iriarte-Mesa et al., 2023). Another study investigated the uptake and translocation of polyacrylic acid (PAA)-coated Ag and FeO NMs. Although both types were taken up by cells, only FeO particles could cross the *in vitro* barrier. This suggests the core material of the NM, not the coating, plays a crucial role in translocation (Mittag et al., 2022b). Furthermore, ingested NMs might undergo complex transformations with the dynamic physicochemical environment of the gastrointestinal tract, which significantly influences their interaction and uptake by the biological system. Consequently, accurately modeling the food matrix effect and the unique GIT exposure conditions is essential for comprehensive NM bio-interaction studies (DeLoid et al., 2017; Antonello et al., 2022).

#### 3.4.2 Systems

The differentiated Caco-2 model and the triculture system Caco-2/HT29-MTX or Caco-2-TC7/HT29-MTX represent the traditional co-culture model to investigate the intestinal barrier functionality *in vitro* (Haddad et al., 2023). Discrepancies between studies can also be attributed to variations in the timing and method of NM administration within the transwell system, specifically whether the NM was added to the apical or basolateral compartment. The commonly used Caco-2 cell line, primarily employed for assessing cell viability, contributes to inconsistencies in observed biological effects and dose–response relationships. This is because different cell types can exhibit varying sensitivities to NMs. For instance, Antonello et al. (2022) also tested primary non-transformed intestinal epithelial cells, HCoEpiC, and the colon cancer-derived HCT116 cell line, in addition to proliferative Caco-2, and observed the cytotoxic effect of FeO NMs in all cell lines with different grades of sensitivity (primary non-transformed cells > Caco-2 and HCT116 cells).

## 4 Discussion

The EFSA acknowledges the importance of AOPs in risk assessment. Their guidance document on NMs emphasizes the role of AOPs in providing a deeper mechanistic understanding of potential human health impacts. Furthermore, using AOPs alongside IATAs can streamline future testing of NMs in food, facilitating the weight-of-evidence approach and minimizing further animal studies. However, while AOP-Wiki is a valuable resource for how NMs can impact organs like the lungs, liver, and reproductive system, information on their effects on the intestine is scarce. This causes a knowledge gap and creates challenges in understanding the relevance of the *in vitro* assays needed for the establishment of IATAs, as described in Step 2 of the nano-guidance document. Scientific evidence documented in the literature demonstrates that internalized NMs can disrupt the normal function of intestinal epithelial cells, and this paves the way for suggesting additional AOPs to elucidate the potential mechanisms through which NMs used in the food sector might exert adverse effects on the gut. By building upon existing AOPs with cytotoxicity as a key event, we developed a novel AOP, AOP 530, and incorporated it into AOP-Wiki. This AOP specifically addresses the potential disruption of the intestinal barrier by food-borne NMs. The presented AOP hypothesized that the endocytosis of specific NMs acts as the MIE for intestinal barrier dysfunction. A critical KE within this pathway is the cytotoxicity observed in enterocytes and goblet cells. These cell types contribute to the establishment of the barrier by expressing TJs relevant to the barrier function. However, it is crucial to acknowledge the inherent complexity of cellular responses to NMs, which often exhibit a non-linear and interconnected nature, rather than following a strictly sequential order. By reviewing the scientific literature, we investigated the documented biological effects of five NMs on the intestinal epithelium often used in the food sector.

### 4.1 Endocytic uptake as a molecular initiating event

Endocytosis is of particular interest in nanotoxicology due to its responsibility for the Trojan horse effect, in which NMs facilitate the internalization of contaminants or serve as precursors, releasing breakdown products that would otherwise exhibit limited cellular permeability. This holds true for several metal oxide NMs. Upon internalization, these NMs can undergo degradation, leading to the uncontrolled release of metal ions and bypassing the regulatory mechanisms associated with metal transporters. In our study, we could reveal that relevant NMs for the food sector can be taken up by intestinal cells *in vitro*.

### 4.2 Endocytosis of NMs can lead to lysosomal disruption (KER1775)

In our study, three of the analyzed NMs (ZnO, CuO, and Ag NMs) are known to release ions after accumulating in the acidic environment of lysosomal vesicles. The ionic forms Zn^2+^, Fe^2+^, Ag^+^, or Cu^2+^ can disrupt the stability of the lysosomal membrane through interactions with the phospholipid bilayer, altering its structure or suppressing enzyme activities involved in maintaining the lysosomal integrity (Wang et al., 2018). The resulting leakage of digestive enzymes can further damage the cellular components and trigger cell death. In addition, an overload of positive ions in lysosomes also triggers anionic and water influx, ultimately leading to a disruption of the lysosome. In addition, the NM itself can exhibit cationic characteristics depending on the surface of the nanoparticles and the specific environments. Such characteristics are described for FeO and SiO_2_ NMs that can lead to an influx of anions and water (proton sponge effect). Lysosomes can swell and lead to the disruption of the lysosomal membrane (Wu et al., 2017; Lee and Hong, 2020), leading to lysosomal dysfunction. However, none of the investigated studies measured the disruption of lysosomal structures in enterocytes or goblet cells, preventing us from establishing a direct link between endocytosis and lysosomal disruption, as described in KER1775 for other NMs.

### 4.3 Lysosomal disruption leads to mitochondrial dysfunction (KER993)

Our hypothesis proposes that lysosomal disruption triggers the release of lysosomal proteases, consequently inducing mitochondrial dysfunction, as described in KER993. Mitochondrial dysfunction encompasses a broad spectrum of alterations in both the mitochondrial structure and function. NM-derived ions interfere with redox cycling in mitochondria, leading to elevated ROS production. In addition, physical interactions between the entire NM and mitochondria and reactive groups on the NP surface can trigger ROS generation. This aspect is confirmed by several studies related to Ag NMs (Braeuning et al., 2018; Domenech et al., 2021; Kämpfer et al., 2021; Jia et al., 2020; Rufino et al., 2021; Juling et al., 2017; Böhmert et al., 2012; Botha et al., 2019; Gioria et al., 2018; Oberemm et al., 2016; Chen et al., 2016). As the concentration of NMs/ions increases within a cell (e.g., through accumulation after chronic or high exposure), the likelihood of interactions with cellular components, particularly mitochondria, also increases. Moreover, the production of ROS can initiate a cascade of additional cellular events with an impact on the intestinal barrier including inflammatory responses (Youn and Choi, 2022; Horie and Tabei, 2021). Xu and colleagues described increased ROS production, pro-inflammatory cytokines, and cytotoxicity after treatment with smaller SiO_2_ nanoparticles, affecting signal transduction pathways (Xu et al., 2022). Pathways such as RhoA/ROCK, leading to the disassembly of TJ, inflammatory signaling cascades, and cytoskeleton, can compromise cellular processes crucial for a healthy barrier (Xu et al., 2022; Jia et al., 2020; Chen et al., 2016; González-Mariscal et al., 2008). Such impairments would not automatically lead to cell death but can be of relevance for the intestinal barrier (Ude et al., 2019).

### 4.4 Mitochondrial dysfunctions lead to cytotoxicity (KER363)

The causal relationship between mitochondrial dysfunction and cell death/injury is well-captured within KER363 in the OECD-endorsed AOP48 and AOP144. ROS plays a critical role in cell death, particularly at higher concentrations. A lower level of ROS production can act as a signaling molecule, even enhancing cellular stress resistance. However, when ROS production overwhelms the cell’s antioxidant defenses, a state of oxidative stress occurs, promoting cell death. Physicochemical properties, the susceptibility of different cell types, and exposure times, are other parameters that can influence different cytotoxicity results. Increased lipid vacuoles and changes in morphologic ultrastructure, like villi and organelles dimensions, have also been observed in NM-treated cells (Li et al., 2023). Moreover, altered expressions of cytoskeleton- and lipid-metabolism-related proteins have also been found in *in vivo* studies (Reichardt et al., 2009; Reichardt et al., 2012). Passive lipid uptake could be progressively normalized through a decrease in the intestinal adsorptive area because of changes in the microvilli length, width, and density of enterocytes. All these changes can contribute to membrane instability, lipid accumulation, oxidative stress, and inflammation, which ultimately lead to cell death machinery activation and intestinal epithelium homeostasis dysregulation (Fang et al., 2021; Anto and Blesso, 2022). The balance between proliferation and apoptosis of intestinal cells is dependent on the microenvironment and stress factors; the defect in balance is strongly connected with several intestinal diseases and GI injuries (Nunes et al., 2014; Subramanian et al., 2020).

### 4.5 Intestinal cell death leads to intestinal barrier disruption (KE3197)

The intestinal barrier is a multilayer system. Alteration of one or more layers of the intestinal barrier leads to increased intestinal permeability and decreased barrier function (Horowitz et al., 2023). Some studies have reported the role of TJs in regulating epithelial cell proliferation. For instance, overexpression of claudin-2 in human colon cells increased proliferation *in vitro* and accelerated tumor growth *in vivo* (Buhrmann et al., 2015; Abu-Farsakh et al., 2017). Moreover, it is known that pro-inflammatory cytokines such as TNF and IL1β or LPS can induce barrier loss by internalization of occludin (Nighot et al., 2017; Kaminsky et al., 2021). The administration of recombinant IL-13 in mice increases claudin-2 expression and augments intestinal paracellular cation permeability (Raju et al., 2020). The causal link between damaged/dying enterocytes and goblet cells leads to increased paracellular permeability due to disruption of the epithelial monolayer and regulation of mucus secretion and thickness; this process, well described in the literature (Horowitz et al., 2023; Paone and Cani, 2020), is now captured within KER3197 in AOP-Wiki.

Other pathophysiologic events leading to barrier disruption can occur in the gut after NM ingestion. For instance, some food additives have been shown to induce microbiota composition alterations. These microbiota alterations have been associated with a reduced mucus layer thickness and an increased gut penetrability linked with intestinal inflammation and metabolic alterations of the intestinal barrier (Paone and Cani, 2020; Chassaing et al., 2017; Viennois and Chassaing, 2018).

## 5 Conclusion

This study proposes a novel AOP, linking NM uptake to intestinal barrier disruption. Although the proposed mechanism is biologically plausible, the available evidence, primarily derived from studies on ZnO, CuO, FeO, SiO_2_, and Ag NMs, offers limited support. The observed variability in study outcomes can be attributed to the heterogeneity in NM properties, biological systems, treatment type, and doses. To strengthen the AOP, further research is required, including systematic investigations of the proposed KER using well-characterized stressors. Identifying a prototypical stressor would also enhance the AOP’s utility for quantitative assessments. It is essential to consider that other AOPs, such as those involving inflammation, may also contribute to intestinal barrier impairment induced by NMs. A comprehensive understanding of NM toxicity requires the integration of multiple AOPs. Despite these limitations, the proposed AOP provides a valuable framework for understanding the potential toxicity of existing and emerging food NMs, guiding future research and risk assessment efforts.

## Data Availability

The original contributions presented in the study are included in the article/supplementary material; further inquiries can be directed to the corresponding author.

## Glossary

AAS: atomic absorption spectrometry
AB-PAS: Alcian blue-PAS staining
AFM: atomic force microscopy
AP: apical treatment in transwell
BL: basolateral treatment in transwell
CBMN: cytokinesis-block micronucleus
CLSM: confocal laser scanning microscopy
CPE: cloud point extraction
CTB: CellTiter-Blue
D: differentiated Caco-2
EDS: energy dispersive X-ray spectroscopy
FCMN: flow cytometric micronucleus
G: cell culture on a glass slide
GSEA: gene set enrichment analysis
H&E: hematoxylin–eosin staining
IBCA: impedance-based cellular assays
IBM: ion beam microscopy
ICP-AES: inductively coupled plasma–atomic emission spectroscopy
ICP-MS: inductively coupled plasma–mass spectrometry
ICP-OES: inductively coupled plasma–optical emission spectrometry
IF: immunofluorescence
IHC: immunohistochemistry
LM: light microscopy
LY: luciferase yellow
NTA: nanoparticle track analysis
Papp: apparent permeability
PL: proliferative Caco-2
PT: cell culture in plate
SEM: scanning electron microscopy
T: cell culture in a transwell system
TEER: transepithelial resistance
TEM: transmission electron microscopy
WB: Western blot
